# 417. COVID-19 Aerostudy: Evaluation of SARS-CoV-2 Virus in the Air of Patients Hospitalized with COVID-19

**DOI:** 10.1093/ofid/ofab466.617

**Published:** 2021-12-04

**Authors:** Hamed Hamza, Margaret Seitsema, Lorraine Conroy, Alfredo J Mena Lora, Eric Wenzler, Scott Borgetti, Benjamin Ladner, Tracy Cable, ashley Dahlquist, Nahed Ismail, Steven Fisher, Taha Ali, Dagmar Sweeney, Susan C Bleasdale

**Affiliations:** 1 UIC School of Public Health, Chicago, Illinois; 2 University of Illinois at Chicago, Chicago, Illinois; 3 University of Illinois Hospital, Chicago, Illinois

## Abstract

**Background:**

At the onset of the COVID-19 pandemic, hospitals implemented infection control measures with limited data on predictors of nosocomial SARS-CoV-2 transmission. We aimed to quantify SARS-CoV-2 presence in an inpatient setting to understand nosocomial risk.

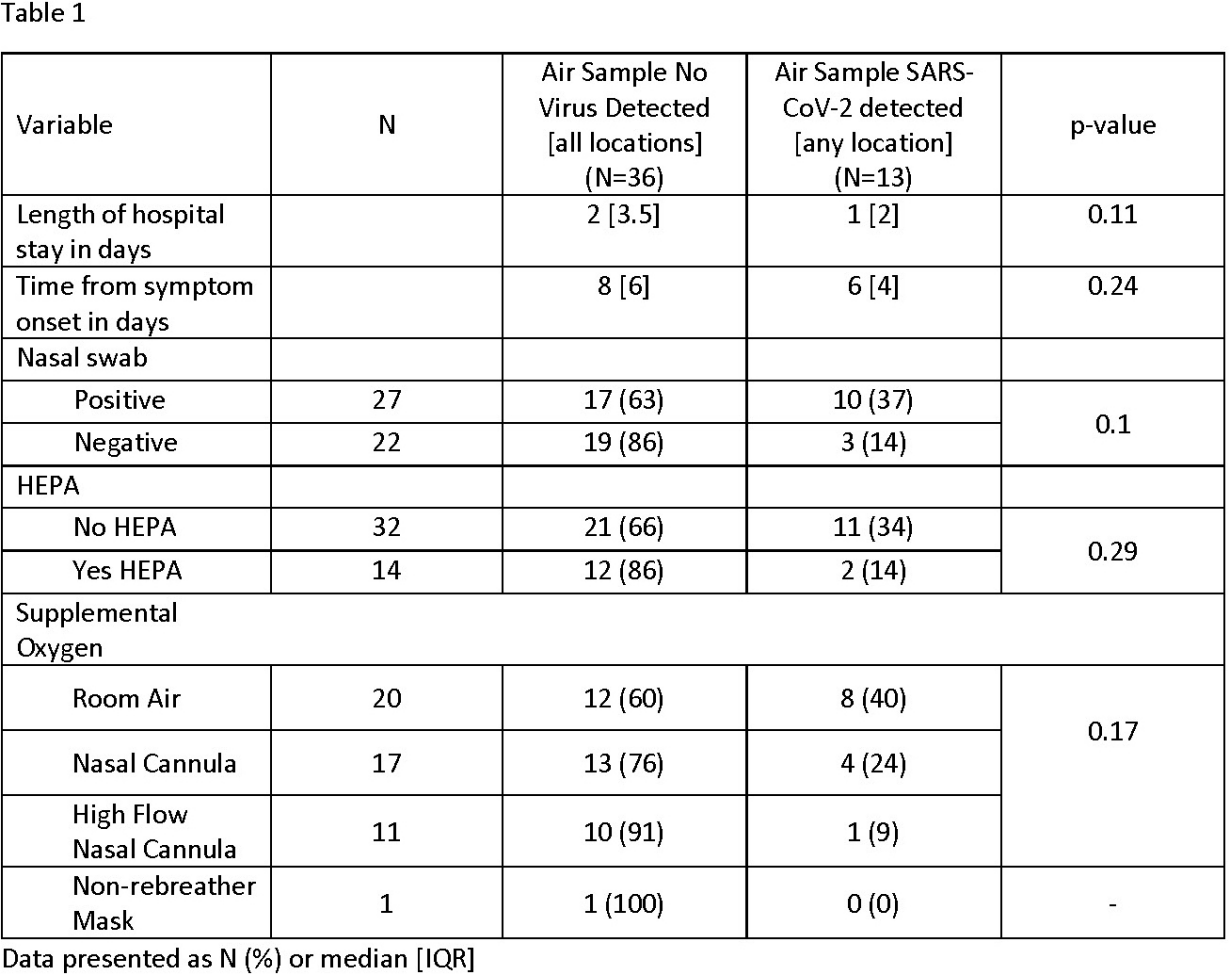

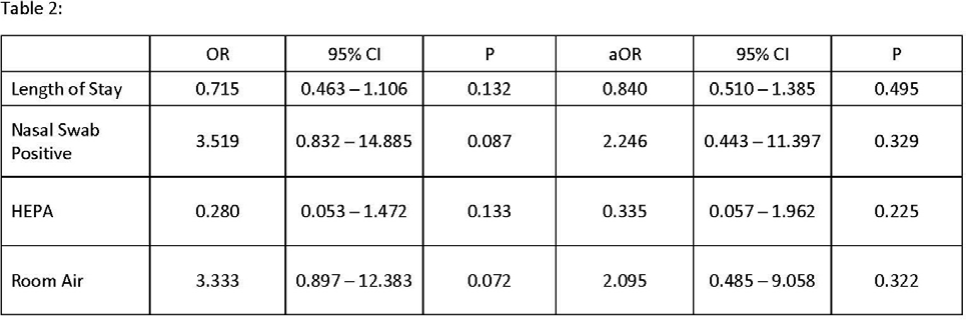

**Methods:**

Patients admitted with confirmed SARS-CoV-2 infection at an urban academic hospital were enrolled. Demographic/clinical characteristics, a PCR nasal swab(NS), and air samples on filter media in the near- (< 6ft) and far-field ( >6ft) of each patient for 3.5 hours were collected. PCR was used to detect SARS-CoV-2 on filter media. Associations between clinical characteristics and presence of SARS-CoV-2 in air samples used Fisher’s exact and Wilcoxon rank sum tests.

**Results:**

Of 52 subjects, 46% had no detectable virus by nasal swab on the day of sampling. Of 104 room air samples, 16% had detectable virus from 25% of rooms, including 10 near and 7 far field samples. Subjects with a positive room air sample had fewer days from symptom-onset compared with those with a negative air sample (median 6 vs. 8, p=0.24). Being on room air and having a nasal swab positive increased the odds of detecting virus in air samples but were not statistically significant.

**Conclusion:**

A small number of air samples with detectable SARS-CoV-2 may suggest lower nosocomial risk than previously anticipated. Multiple subject and environmental factors may have contributed to this finding including patient source control masking, anti-viral therapies and HEPA filtration. The decreased association of virus in the air of those with more days of symptoms but with the need for supplemental oxygen may be related to what is now known about the COVID-19 inflammatory response after the infectious period.

**Disclosures:**

**All Authors**: No reported disclosures

